# On new systems of rabies virus using vaccination variable with modification

**DOI:** 10.1016/j.mex.2025.103308

**Published:** 2025-04-09

**Authors:** Ibtehal Alazman

**Affiliations:** Department of Mathematics and Statistics, College of Science, Imam Mohammad Ibn Saud Islamic University (IMSIU), Riyadh, Saudi Arabia

**Keywords:** Fractional calculus, Fractal calculus, Rabies virus dynamical system, Fractal-fractional calculus

## Abstract

Usually, a compartmental epidemiological modeling such as the SIR (Susceptible-Infectious-Recovered) or SEIR (Susceptible-Exposed-Infectious-Recovered) model must be modified in order to include a vaccine element in a mathematical representation of rabies. This element may influence the dynamics of disease transmission by accounting for vaccinated persons. In this effort, we suggest new two different dynamical systems of rabies virus utilizing the vaccination as a variable with modification in fractal-fractional calculus. Analysis of data is investigated with several examples. Moreover, the solvability of fractal-fractional dynamic system is given using the two dimensional and three dimensional fractal-fractional differential operators. The solution is suggested by utilizing the modified fractal-fractional integral operator. Finally, we estimate the greatest number of infectious individuals and the time at which this peak occurs in order to examine the peak infection in every scenario. The outcomes for the Baseline case and the altered beginning conditions will be computed and shown. The methodology of this paper is as follows:

• Include a vaccine element, as a variable to get five dimensional ordinary system with vaccination (RVT-Vaccination);
• Generalize the above system RVT-Vaccination by using the most recent fractal-fractional differential operators, with two and three fractal-fractional powers.
• Analyze the three systems and establish the solvability.

## Graphical abstract

**Figure:**
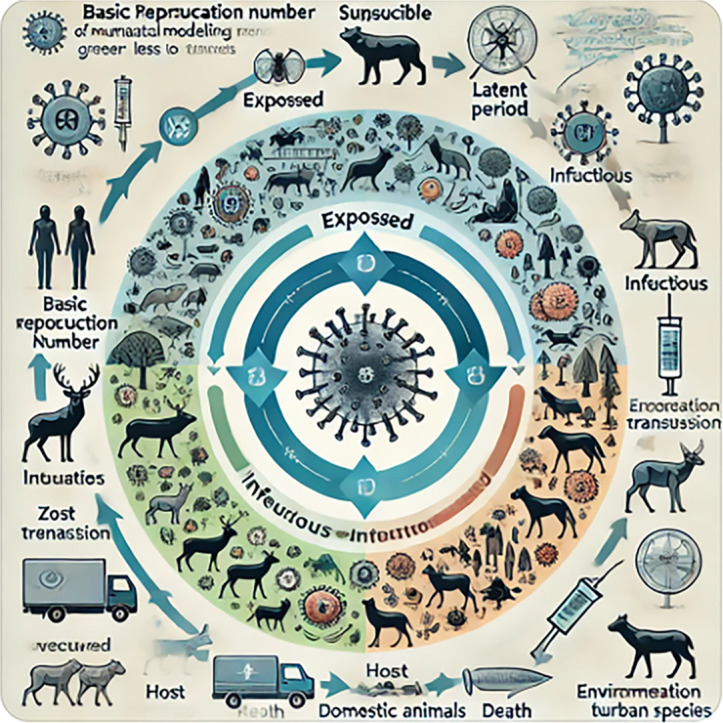

Specifications table

**Table:** 

Subject area:	Mathematics and Statistics
More specific subject area:	*Applied Mathematics*
Name of your method:	*Fractal-fractional calculus*
Name and reference of original method:	[11] Atangana, Abdon. ``Fractal-fractional differentiation and integration: connecting fractal calculus and fractional calculus to predict complex system.'' Chaos, solitons & fractals 102 (2017): 396–406.
Resource availability:	*Not applicable.*

## Background

Understanding the dynamics of rabies virus transmission (RVT), forecasting outbreaks, and developing control measures all depend on mathematical modeling of the virus. Some of the simplest models for infectious diseases is SIR, which has been modified for rabies by adding a death class because of the disease's high fatality rate. The exposed (latent) phase of rabies is included in SEIR. Stochastic models that take into account incidents at random are frequently significant, even if they may only involve a small number of cases (particularly in species). The dispersal of linked populations (such as wildlife in various geographic locations) can be explained using Meta population systems. Fractional case of RVT is studied recently by many researchers using the classical and modify types of fractional operators (see [[1], [2], [3], [4], [5]]).

The dynamics of infectious diseases are frequently modeled using differential equations (ODEs), although some of the intricacies of real-world processes may be missed by ODEs. Fractal-fractional models (FFMs), on the other hand, offer a more sophisticated method by combining the ideas of fractal geometry and fractional calculus. By permitting the maximum infection rate to fluctuate across time or space, fractal-fractional models are better able to represent the complex and non-linear dynamics of infection transmission. The fractional-order dynamics of disease transmission or the fractal character of interaction networks could be the cause of this diversity. The fractal character of disease dynamics can be included into fractal-fractional models, which enable variable and perhaps long-tailed infectious period distributions. A more accurate portrayal of the persistence and return of disease could come from this (see [[6], [7], [8], [9], [10]]). By adding non-integer derivatives, fractional calculus enables us to generalize the dynamics of infectious disease models such as the SEIR-V model. Memory effects and anomalous diffusion, which are frequently observed in real-world systems like the spread of illness, can be better captured by this method (see [11,12]).

We propose two novel dynamical systems of the rabies virus in this endeavor, considering the vaccination as a variable that is modified via fractional calculus. Data analysis is examined using a number of cases. Furthermore, the fractal-fractional operators are used to determine the resolution of fractional dynamic systems. Section 2 describes the method that we consider; Section 3 is about FFMs of RVT. Limitations and challenges are given in Section 4.

## Method details

### *RVT-Vaccination system*

We suggest the following ordinary system with vaccination

(1) $$\begin{array}{ccc} & & S^{\cdot }=-\rho SI-\upsilon S\\ & & E^{\cdot }=\rho SI-\varrho E\\ & & I^{\cdot }=\varrho E-\sigma I\\ & & R^{\cdot }=\sigma I\\ & & V^{\cdot }=\upsilon S, \end{array}$$

where ρ is the transmission rate, υ is the vaccination rate, ϱ is the rate at which infectious exposure progresses and σ is the recovery rate. Effects of Immunization are as follows: By reducing the number of susceptible people, the vaccine term υS lowers the likelihood that the infection will spread. If vaccination rates are high enough, the illness could possibly be completely eradicated or its propagation may be considerably held down. To simulate this system, we need initial conditions for each compartment and parameter values. Next, we consider a set of simulations of System

Example 1 Consider the following data

• Initial population: 1000 individuals.
• Initial susceptible population: 990 (most are susceptible).
• Initial exposed population: 5 (few are exposed).
• Initial infectious population: 5.
• Vaccination rate υ=0.02 (2 % of susceptible individuals are vaccinated per time unit).
• Transmission rate ρ=0.5
• Exposed to infectious progression rate ϱ=0.25
• Recovery or death rate σ=0.1

**Fig. 1:**
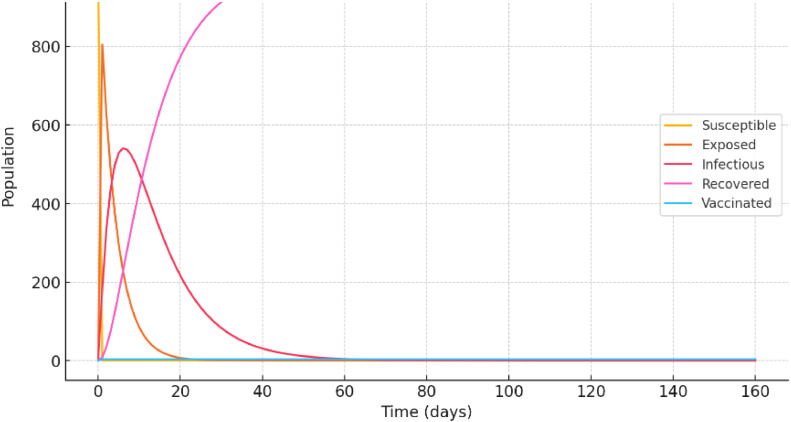
The SEIR-V simulated model for rabies with vaccination. The populations for every compartment (Susceptible, Exposed, Infectious, Recovered, and Vaccinated) are depicted in the illustration as they evolve over time.

Fig. 1 indicates that as people are exposed to the virus or receive vaccinations, the susceptible population (S) gradually declines. As new people become infected, the exposed population (E) first increases, yet as they become infected, it decreases. As more people recover or pass away, the infectious population (I) peaks and then gradually declines. As people recover, the Recovered population (R) grows. As more vulnerable people are vaccinated, the Vaccinated population (V) gradually increases. When vaccine is introduced, the percentage of susceptible people is drastically reduced, which lessens the infection's general impact.

**Fig. 2:**
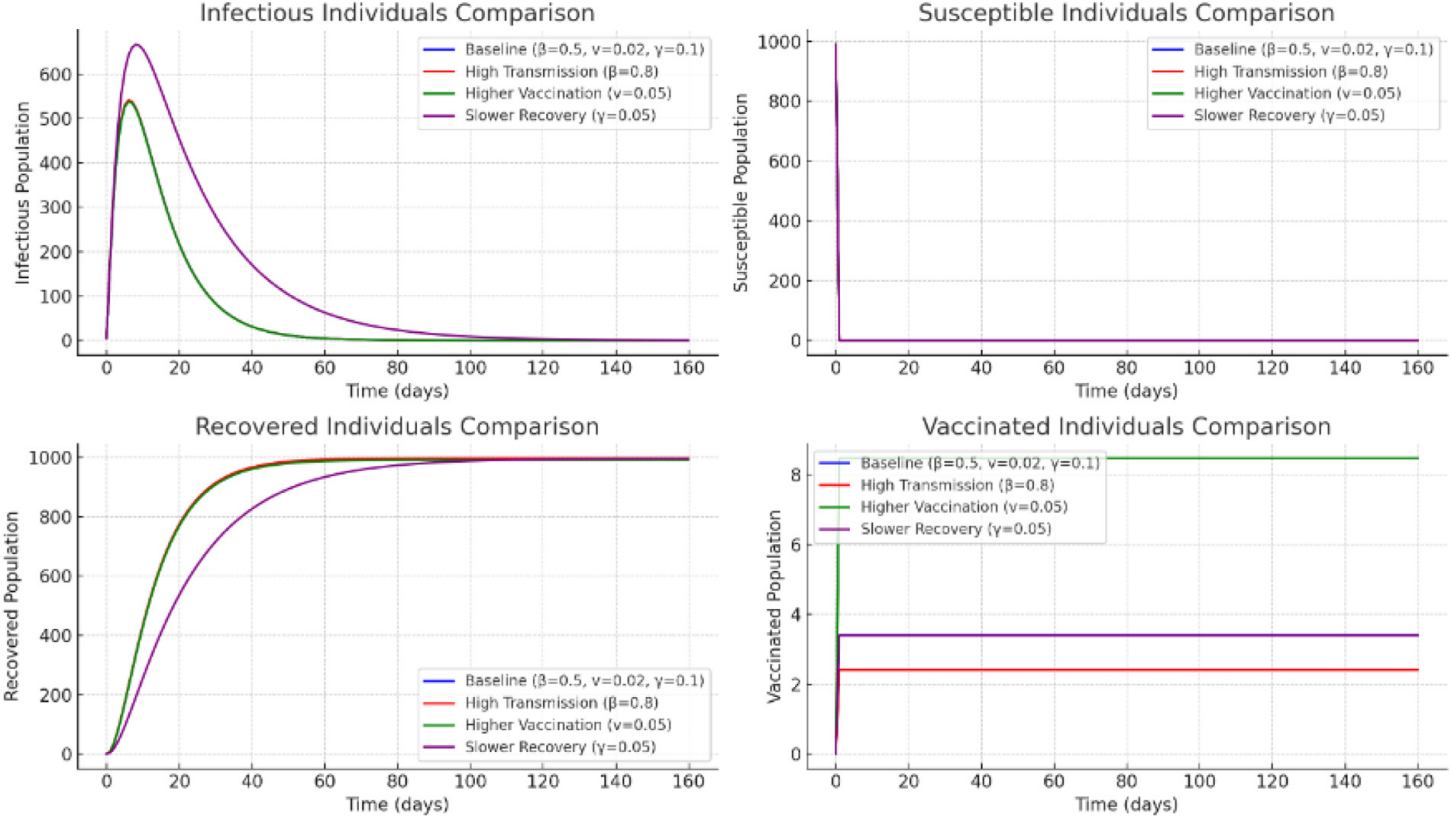
The charts display the results of four distinct settings in Example 2.

**Fig. 3:**
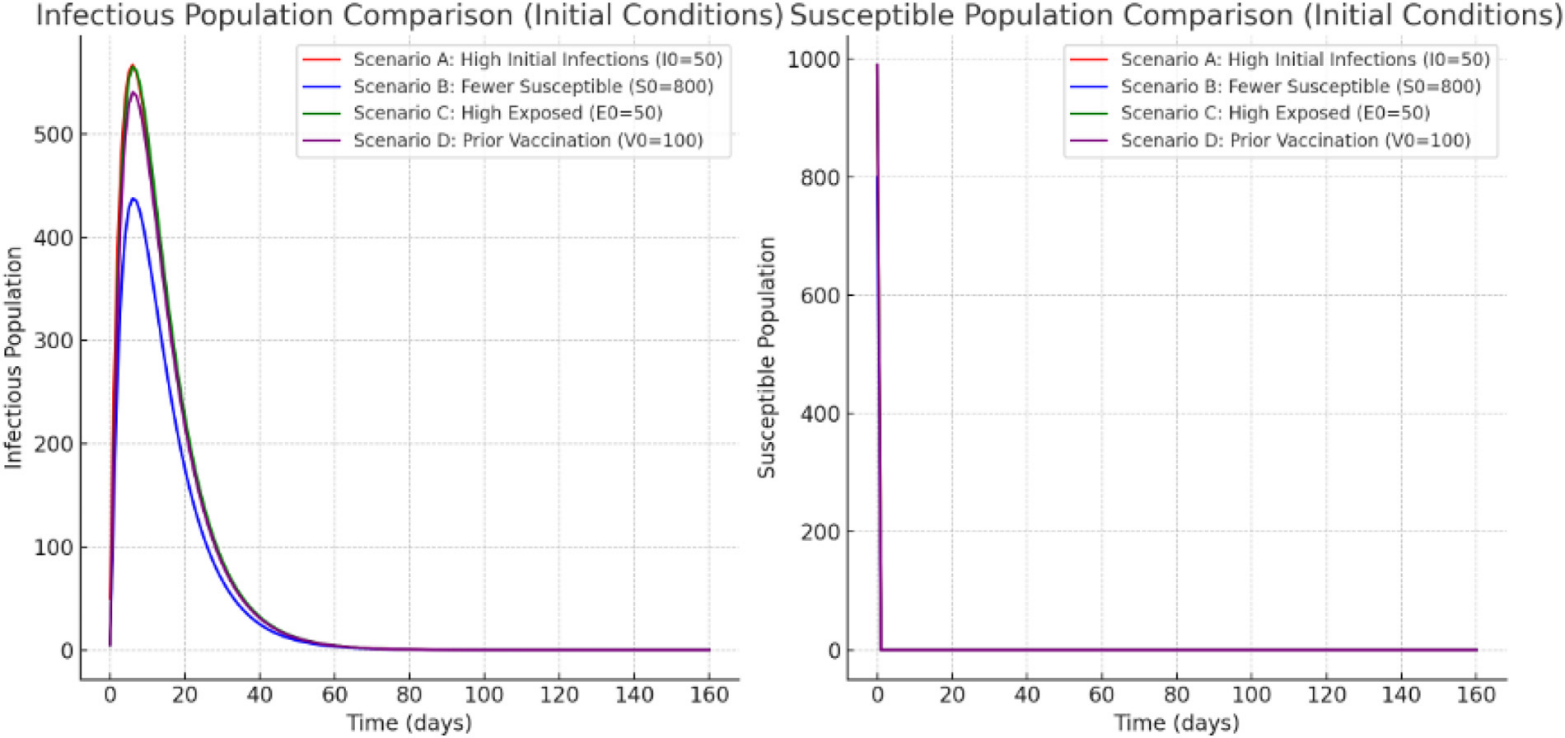
The charts display the results of four distinct settings in Example 3 with respect to the initial conditions.

Example 2 Assume the following data:

• Line: (ρ,υ,σ)=(0.5,0.02,0.1);
• High transmission: (ρ,υ,σ)=(0.8,0.02,0.1);
• High Vaccination: (ρ,υ,σ)=(0.5,0.05,0.1);
• Low Recovery: (ρ,υ,σ)=(0.5,0.02,0.05);

Fig. 2 shows different cases: High Transmission: A greater transmission rate ρ causes a quicker peak in infectious cases and a quicker decline in the number of vulnerable people. Increased immunization: Raising the immunization rate υ protects more people at an earlier age by boosting the amount of immunized individuals and drastically lowering the amount of infected persons. Slower Recovery: A lower recovery rate σ causes the infected population to remain larger for a longer amount of time by slowing down the decline in infectious episodes (Fig. 3).

Example 3 In this example, we have selected other initial conditions. We have the following data:

• To replicate a more severe outbreak, increase the initial number of infectious persons ($I_{0}$) from 0 to 50 instead of 5.
• Considering that some members of the general public possess the immune, reduce the starting number of vulnerable people ($S_{0}$) from 0 to 800.
• To simulate a larger latent populace, increase the starting total amount of exposed individuals ($E_{0}$) by 0 to 50.
• To examine the effects of earlier vaccination campaigns, begin with 100 vaccinated people ($V_{0}$).

### *Fractal-fractional RVT-Vaccination system*

In this section, we have presented a generalization of System (1) by using the following definition.

Definition 1 Consider the differentiable function g(x) over (0, b). The following is the definition of the Riemann-Liouville fractal-fractional differential operator [13]:

$${}^{ARL}D_{t}^{\alpha ,\beta }g\left(t\right)≔\frac{1}{\Gamma \left(n-\alpha \right)}\frac{d}{dt^{\beta }}\int_{0}^{t}g\left(\tau \right){\left(t-\tau \right)}^{n-\alpha -1}d\tau \;\alpha ,\beta \in \left(n-1,n\right],$$

where

$$\frac{dg\left(t\right)}{dt^{\beta }}={\text{lim}}_{t\to \tau }\frac{g\left(t\right)-g\left(\tau \right)}{t^{\beta }-\tau^{\beta }}.$$

Three fractal-fractional factors are considered in the next abstraction:

$${}^{ARL}D_{t}^{\alpha ,\beta ,\gamma }g\left(t\right)≔\frac{1}{\Gamma \left(n-\alpha \right)}\frac{d^{\gamma }}{dt^{\beta }}\int_{0}^{t}g\left(\tau \right){\left(t-\tau \right)}^{n-\alpha -1}d\tau ,\;\alpha ,\beta ,\gamma \in \left(n-1,n\right],$$

where

$\frac{d^{\gamma }g\left(t\right)}{dt^{\beta }}=\underset{t\to \tau }{\text{lim}}\frac{g^{\gamma }\left(t\right)-g^{\gamma }\left(\tau \right)}{t^{\beta }-\tau^{\beta }}.$

The formula for the fractal-fractional integral operators of order α,β>0 and α,β,γ>0 are as follows:

$$J_{t}^{\alpha ,\beta }g\left(t\right)≔\frac{\beta }{\Gamma \left(\alpha \right)}\int_{0}^{t}\tau^{\alpha -1}g\left(\tau \right){\left(t-\tau \right)}^{\alpha -1}d\tau ,\;\alpha ,\beta >0;$$

and

$$J_{t}^{\alpha ,\beta ,\gamma }g\left(t\right)≔\frac{\beta }{\gamma \Gamma \left(\alpha \right)}\int_{0}^{t}\tau^{\alpha -1}g\left(\tau \right){\left(t-\tau \right)}^{\alpha -1}d\tau ,\;\alpha ,\beta ,\gamma >0,$$

correspondingly.

Based on the above definition, we investigate the next fractional-fractal system

(2) $$\begin{array}{ccc} & & {}^{ARL}D_{t}^{\alpha ,\beta }S\left(t\right)=-\rho S\left(t\right)I\left(t\right)-\upsilon S\left(t\right)\\ & & {}^{ARL}D_{t}^{\alpha ,\beta }E\left(t\right)=\rho S\left(t\right)I\left(t\right)-\varrho E\left(t\right)\\ & & {}^{ARL}D_{t}^{\alpha ,\beta }I\left(t\right)=\varrho E\left(t\right)-\sigma I\left(t\right)\\ & & {}^{ARL}D_{t}^{\alpha ,\beta }R\left(t\right)=\sigma I\left(t\right)\\ & & {}^{ARL}D_{t}^{\alpha ,\beta }V\left(t\right)=\upsilon S\left(t\right). \end{array}$$

Similarly, we can consider the 3D fractal-fractional operator

(3) $$\begin{array}{ccc} & & {}^{ARL}D_{t}^{\alpha ,\beta ,\gamma }S\left(t\right)=-\rho S\left(t\right)I\left(t\right)-\upsilon S\left(t\right)\\ & & {}^{ARL}D_{t}^{\alpha ,\beta ,\gamma }E\left(t\right)=\rho S\left(t\right)I\left(t\right)-\varrho E\left(t\right)\\ & & {}^{ARL}D_{t}^{\alpha ,\beta ,\gamma }I\left(t\right)=\varrho E\left(t\right)-\sigma I\left(t\right)\\ & & {}^{ARL}D_{t}^{\alpha ,\beta ,\gamma }R\left(t\right)=\sigma I\left(t\right)\\ & & {}^{ARL}D_{t}^{\alpha ,\beta ,\gamma }V\left(t\right)=\upsilon S\left(t\right). \end{array}$$

Fractal-fractional systems enable higher precise and adaptable modeling of intricate, memory-rich systems in a variety of fields by combining the advantages of fractional calculus and fractal geometry. Fractal-fractional systems enable more precise and adaptable modeling of intricate, memory-rich systems in a variety of fields by combining the advantages of fractional calculus and fractal geometry. Fractal-fractional systems make it possible to concurrently model phenomena at several scales. When processes at several scales (including the human being, community, and demographic stage) interact, this is particularly helpful in multi-scale systems. For instance, fractional systems are better at capturing interactions at tiny scales (individual organisms) that impact larger population dynamics than typical models used in ecology or epidemiological (see [[14], [15], [16], [17]]).

Fractal-fractional models are particularly well-suited for simulating the spread of rabies because they combine the advantages of fractal geometry and classical fractional models: Memory and Heterogeneous Space: A more realistic depiction of the temporal and spatial features of rabies transmission is made possible by the combination of memory effects and fractal structures. Anomalous Diffusion: The erratic movement patterns of rabid animals are an example of anomalous diffusion processes, which are naturally included in fractal-fractional models. Improved Predictive Power: These algorithms increase the precision of predictions on the spread of disease by accounting for the intricate topology of animal interactions and movement. Variability: Able to fit particular data sets by varying the fractal dimension and fractional derivative order to mimic a variety of biological systems For instance applications, we refer to the efforts in [[18], [19], [20]].

Example 4 Let α=0.8 and β=1 with the initial conditions set $\left(S_{0},E_{0},I_{0},R_{0},V_{0}\right)=\left(990,5,5,0,0\right)$ and the parameters set (ρ,ϱ,σ,υ)=(0.5,0.25,0.1,0.02).

**Fig. 4:**
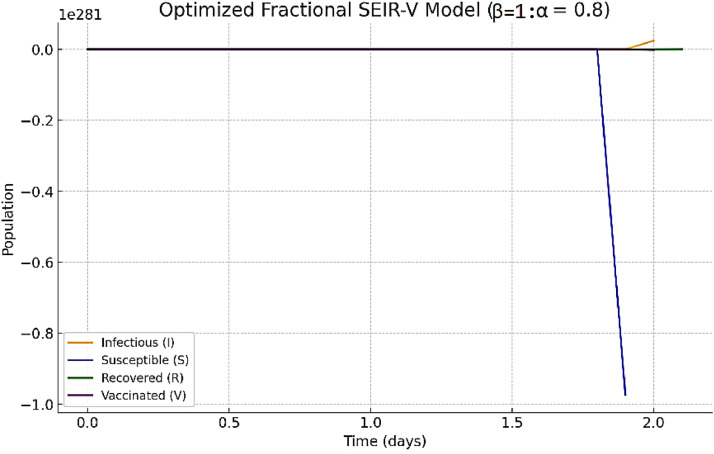
The optimization of fractal-fractional system in Example 4, with total time t=100 and h=0.1 and α=0.8,β=1 (fractional case).

Fig. 4 presents the optimized System by using the direct fractional differentiation matrix method. This model will demonstrate the impact of adding a fractional order α on disease spread. The system has stronger ``memory'' when α is less, which means that its previous dynamics have a greater impact on its present state. Spatial Heterogeneity: Fragmented landscapes with fractal features are where rabies is spread in wildlife, such as foxes and bats. While fractal-fractional models directly include this variability, integer-order models are unable to do so.

Latency and Long-Term Memory: Rabies has a protracted incubation period, and knowledge of memory effects is essential to comprehending the disease's long-term spread. These long-term relationships can be accurately simulated by fractional and fractal-fractional models.

**Fig. 5:**
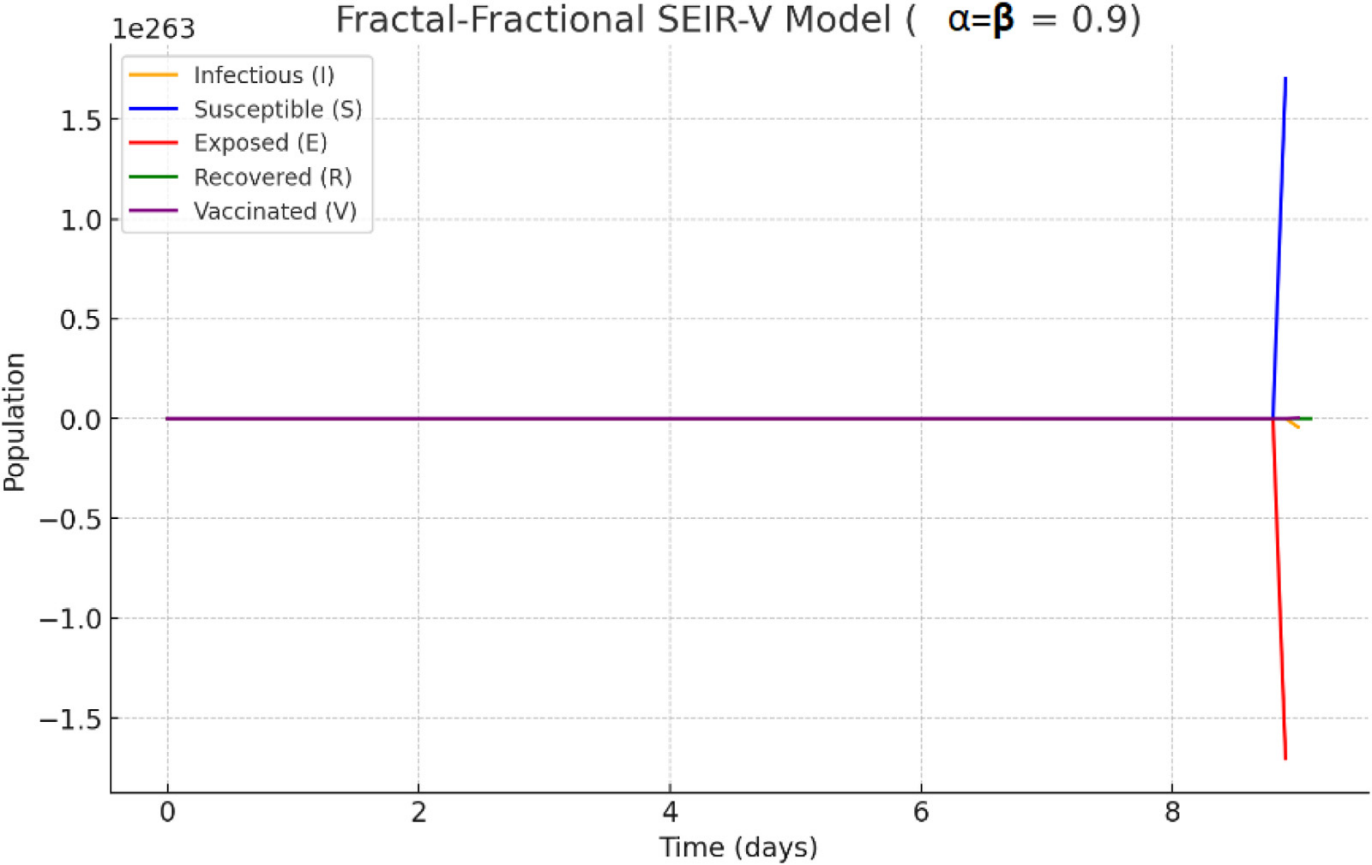
The optimization of fractal-fractional system in Example 4 with (α,β)=(0.9,0.9).

Under the same set of parameters, with α=β=0.9, Fig. 5 shows the optimization of the fractal-fractional system in (2). The impact of memories is slightly augmented by this modification, which means that while the dynamics will still have some fractional characteristics, they will be closer to classical derivatives (α=1.0). Since the method takes into account the effects of previous states, the impact of vaccination may look more gradual in reducing the population at risk when α=0.9.

The particulars of the epidemic and the environment you're attempting to portray will determine whether you use a fractional SEIR-V model or a fractal-fractional SEIR-V model. We proceed to examine each one's benefits and distinctions. The memory effects of illness progression are captured by the fractional model, which has advantages. Excellent for illnesses when exponential distributions do not adequately reflect the time frame of infection, incubation, or recovery (i.e., time since infection matters). Although the restrictions unless further changes are made, it is unable to reflect spatial complexity, or variability in surroundings or demographic. The fractal-fractional system provides a more comprehensive view of how diseases spread in particular habitats by modeling both temporal effect of memory and spatial complexity. Moreover, it is helpful in comprehending the transmission of disease in areas that are fragmented, diverse, or fractal in nature.

## Method validation

### *Solvability of system*

The integral system corresponds to System (2) is

(4) $$\begin{array}{ccc} & & S\left(t\right)=S_{0}-J^{\alpha ,\beta }\left(\rho S\left(t\right)I\left(t\right)-\upsilon S\left(t\right)\right)\\ & & E\left(t\right)=E_{0}+J^{\alpha ,\beta }\left(\rho S\left(t\right)I\left(t\right)-\varrho E\left(t\right)\right)\\ & & I\left(t\right)=I_{0}+J^{\alpha ,\beta }\left(\varrho E\left(t\right)-\sigma I\left(t\right)\right)\\ & & R\left(t\right)=R_{0}+\sigma J^{\alpha ,\beta }I\left(t\right)\\ & & V\left(t\right)=V_{0}+\upsilon J^{\alpha ,\beta }S\left(t\right). \end{array}$$

Equivalently,

(5) $$\begin{array}{ccc} & & S\left(t\right)=S_{0}-\frac{\beta }{\Gamma \left(\alpha \right)}\int_{0}^{t}\tau^{\alpha -1}\left(\rho S\left(\tau \right)I\left(\tau \right)-\upsilon S\left(\tau \right)\right){\left(t-\tau \right)}^{\alpha -1}d\tau \\ & & E\left(t\right)=E_{0}+\frac{\beta }{\Gamma \left(\alpha \right)}\int_{0}^{t}\tau^{\alpha -1}\left(\rho S\left(\tau \right)I\left(\tau \right)-\varrho E\left(\tau \right)\right){\left(t-\tau \right)}^{\alpha -1}d\tau \\ & & I\left(t\right)=I_{0}+\frac{\beta }{\Gamma \left(\alpha \right)}\int_{0}^{t}\tau^{\alpha -1}\left(\varrho E\left(\tau \right)-\sigma I\left(\tau \right)\right){\left(t-\tau \right)}^{\alpha -1}d\tau \\ & & R\left(t\right)=R_{0}+\sigma \frac{\beta }{\Gamma \left(\alpha \right)}\int_{0}^{t}\tau^{\alpha -1}I\left(\tau \right){\left(t-\tau \right)}^{\alpha -1}d\tau \\ & & V\left(t\right)=V_{0}+\upsilon \frac{\beta }{\Gamma \left(\alpha \right)}\int_{0}^{t}\tau^{\alpha -1}S\left(\tau \right){\left(t-\tau \right)}^{\alpha -1}d\tau . \end{array}$$

By letting

$$\begin{array}{ccc} & & W_{1}\left(t\right)≔\left(\rho S\left(t\right)I\left(t\right)-\upsilon S\left(t\right)\right)\\ & & W_{2}\left(t\right)≔\left(\rho S\left(t\right)I\left(t\right)-\varrho E\left(t\right)\right)\\ & & W_{3}\left(t\right)≔\left(\varrho E\left(t\right)-\sigma I\left(t\right)\right)\\ & & W_{4}\left(t\right)≔I\left(t\right)\\ & & W_{5}\left(t\right)≔S\left(t\right), \end{array}$$

System (5) becomes

$$\begin{array}{ccc} & & S\left(t\right)=S_{0}-\frac{\beta }{\Gamma \left(\alpha \right)}\int_{0}^{t}\tau^{\alpha -1}W_{1}\left(\tau \right){\left(t-\tau \right)}^{\alpha -1}d\tau \\ & & E\left(t\right)=E_{0}+\frac{\beta }{\Gamma \left(\alpha \right)}\int_{0}^{t}\tau^{\alpha -1}W_{2}\left(\tau \right){\left(t-\tau \right)}^{\alpha -1}d\tau \\ & & I\left(t\right)=I_{0}+\frac{\beta }{\Gamma \left(\alpha \right)}\int_{0}^{t}\tau^{\alpha -1}W_{3}\left(\tau \right){\left(t-\tau \right)}^{\alpha -1}d\tau \\ & & R\left(t\right)=R_{0}+\sigma \frac{\beta }{\Gamma \left(\alpha \right)}\int_{0}^{t}\tau^{\alpha -1}W_{4}\left(\tau \right){\left(t-\tau \right)}^{\alpha -1}d\tau \\ & & V\left(t\right)=V_{0}+\upsilon \frac{\beta }{\Gamma \left(\alpha \right)}\int_{0}^{t}\tau^{\alpha -1}W_{5}\left(\tau \right){\left(t-\tau \right)}^{\alpha -1}d\tau , \end{array}$$

where $W_{i},i=1,\ldots ,5$ are continuous. Clearly, for t∈[0,T],T<∞, we get

$$\begin{array}{ccc} & & ∥S\left(t\right)∥\leq \left|S_{0}\right|+\frac{\beta }{\Gamma \left(\alpha \right)}∥W_{1}∥\left(\frac{\Gamma {\left(\alpha \right)}^{2}T^{2\alpha -1}}{\Gamma \left(2\alpha \right)}\right)\\ & & ∥E\left(t\right)∥\leq \left|E_{0}\right|+\frac{\beta }{\Gamma \left(\alpha \right)}∥W_{2}∥\left(\frac{\Gamma {\left(\alpha \right)}^{2}T^{2\alpha -1}}{\Gamma \left(2\alpha \right)}\right)\\ & & ∥I\left(t\right)∥\leq \left|I_{0}\right|+\frac{\beta }{\Gamma \left(\alpha \right)}∥W_{3}∥\left(\frac{\Gamma {\left(\alpha \right)}^{2}T^{2\alpha -1}}{\Gamma \left(2\alpha \right)}\right)\\ & & ∥R\left(t\right)∥\leq \left|R_{0}\right|+\sigma \frac{\beta }{\Gamma \left(\alpha \right)}∥W_{4}∥\left(\frac{\Gamma {\left(\alpha \right)}^{2}T^{2\alpha -1}}{\Gamma \left(2\alpha \right)}\right)\\ & & ∥V\left(t\right)∥\leq \left|V_{0}\right|+\upsilon \frac{\beta }{\Gamma \left(\alpha \right)}∥W_{5}∥\left(\frac{\Gamma {\left(\alpha \right)}^{2}T^{2\alpha -1}}{\Gamma \left(2\alpha \right)}\right), \end{array}$$

where ∥.∥ is the sup. norm and all the parameters are positive with α>0,β>0. Suppose that $\bar{W}≔\text{ma}x_{i=1,\ldots ,5}∥W_{i}∥.$ Moreover, assume that κ≔max{1,σ,υ} and $\Sigma ≔(\frac{\Gamma {\left(\alpha \right)}^{2}T^{2\alpha -1}}{\Gamma (2\alpha )})>0.$ Then we get the following inequalities system:

$$\begin{array}{ccc} & & ∥S\left(t\right)∥\leq \left|S_{0}\right|+\frac{\beta \kappa }{\Gamma \left(\alpha \right)}\bar{W}\Sigma \\ & & ∥E\left(t\right)∥\leq \left|E_{0}\right|+\frac{\beta \kappa }{\Gamma \left(\alpha \right)}\bar{W}\Sigma \\ & & ∥I\left(t\right)∥\leq \left|I_{0}\right|+\frac{\beta \kappa }{\Gamma \left(\alpha \right)}\bar{W}\Sigma \\ & & ∥R\left(t\right)∥\leq \left|R_{0}\right|+\frac{\beta \kappa }{\Gamma \left(\alpha \right)}\bar{W}\Sigma \\ & & ∥V\left(t\right)∥\leq \left|V_{0}\right|+\frac{\beta \kappa }{\Gamma \left(\alpha \right)}\bar{W}\Sigma . \end{array}$$

Then the operator $Q:R^{5}\to R^{5},$

$$\begin{array}{ccc} Q\left(S,E,I,R,V\right) & = & (S_{0}-\frac{\beta }{\Gamma \left(\alpha \right)}\int_{0}^{t}\tau^{\alpha -1}W_{1}\left(\tau \right){\left(t-\tau \right)}^{\alpha -1}d\tau ,\\ & & E_{0}+\frac{\beta }{\Gamma \left(\alpha \right)}\int_{0}^{t}\tau^{\alpha -1}W_{2}\left(\tau \right){\left(t-\tau \right)}^{\alpha -1}d\tau ,\\ & & I_{0}+\frac{\beta }{\Gamma \left(\alpha \right)}\int_{0}^{t}\tau^{\alpha -1}W_{3}\left(\tau \right){\left(t-\tau \right)}^{\alpha -1}d\tau ,\\ & & R_{0}+\sigma \frac{\beta }{\Gamma \left(\alpha \right)}\int_{0}^{t}\tau^{\alpha -1}W_{4}\left(\tau \right){\left(t-\tau \right)}^{\alpha -1}d\tau ,\\ & & V_{0}+\upsilon \frac{\beta }{\Gamma \left(\alpha \right)}\int_{0}^{t}\tau^{\alpha -1}W_{5}\left(\tau \right){\left(t-\tau \right)}^{\alpha -1}d\tau ) \end{array}$$

is bounded, continuous and equi-continuous in a ball of radius $r=℘\frac{\beta \kappa }{\Gamma (\alpha )}\bar{W}\Sigma ,$ where $℘≔\text{max}\{|S_{0}\left|,\right|E_{0}\left|,\right|I_{0}\left|,\right|R_{0}\left|,\right|V_{0}|\}.$ Hence, in view of the fixed point theorem, Q has a fixed point, which is unique when Q is a contraction mapping. This can be recognized when $\frac{℘\beta \kappa \Sigma }{\Gamma (\alpha )}<1.$

As a conclusion, we can formulate the following result:

Theorem 1 *Assume System (2) with positive parameters*σ,υ*; and continuous variables*S,E,I,R,V*. Then it has at least one solution in the ball of radius*$r=℘\frac{\beta \kappa }{\Gamma (\alpha )}\bar{W}\Sigma ,$*where*κ≔max{1,σ,υ}*,*$\Sigma =\frac{\Gamma {\left(\alpha \right)}^{2}T^{2\alpha -1}}{\Gamma (2\alpha )},$*and*$℘=\text{max}\{|S_{0}\left|,\right|E_{0}\left|,\right|I_{0}\left|,\right|R_{0}\left|,\right|V_{0}|\}.$*Moreover, if*

$$\frac{℘\beta \kappa \Sigma }{\Gamma \left(\alpha \right)}\langle 1,℘\rangle 0,\alpha >0,\beta >0$$

then the system has a unique solution.

Similarly for System (3), we have the following result:

Theorem 2 *Assume System (3) with positive parameters*σ,υ*; and continuous variables*S,E,I,R,V*. Then it has at least one solution in the ball of radius*$R=℘\frac{\beta \kappa }{\gamma \Gamma (\alpha )}\bar{W}\Sigma ,$*where*κ≔max{1,σ,υ}*,*$\Sigma =\frac{\Gamma {\left(\alpha \right)}^{2}T^{2\alpha -1}}{\Gamma (2\alpha )},$*and*$℘=\text{max}\{|S_{0}\left|,\right|E_{0}\left|,\right|I_{0}\left|,\right|R_{0}\left|,\right|V_{0}|\}.$*Moreover, if*

$$\frac{℘\beta \kappa \Sigma }{\gamma \Gamma \left(\alpha \right)}\langle 1,℘\rangle 0,\alpha >0,\beta >0$$

then the system has a unique solution.

Note that the integral system corresponds to System (3) is as follows:

$$\begin{array}{ccc} & & S\left(t\right)=S_{0}-\frac{\beta }{\gamma \Gamma \left(\alpha \right)}\int_{0}^{t}\tau^{\alpha -1}\left(\rho S\left(\tau \right)I\left(\tau \right)-\upsilon S\left(\tau \right)\right){\left(t-\tau \right)}^{\alpha -1}d\tau \\ & & E\left(t\right)=E_{0}+\frac{\beta }{\gamma \Gamma \left(\alpha \right)}\int_{0}^{t}\tau^{\alpha -1}\left(\rho S\left(\tau \right)I\left(\tau \right)-\varrho E\left(\tau \right)\right){\left(t-\tau \right)}^{\alpha -1}d\tau \\ & & I\left(t\right)=I_{0}+\frac{\beta }{\gamma \Gamma \left(\alpha \right)}\int_{0}^{t}\tau^{\alpha -1}\left(\varrho E\left(\tau \right)-\sigma I\left(\tau \right)\right){\left(t-\tau \right)}^{\alpha -1}d\tau \\ & & R\left(t\right)=R_{0}+\sigma \frac{\beta }{\gamma \Gamma \left(\alpha \right)}\int_{0}^{t}\tau^{\alpha -1}I\left(\tau \right){\left(t-\tau \right)}^{\alpha -1}d\tau \\ & & V\left(t\right)=V_{0}+\upsilon \frac{\beta }{\gamma \Gamma \left(\alpha \right)}\int_{0}^{t}\tau^{\alpha -1}S\left(\tau \right){\left(t-\tau \right)}^{\alpha -1}d\tau . \end{array}$$

Thus, we can define the following operator $P:R^{5}\to R^{5},$

$$\begin{array}{ccc} P\left(S,E,I,R,V\right) & = & (S_{0}-\frac{\beta }{\gamma \Gamma \left(\alpha \right)}\int_{0}^{t}\tau^{\alpha -1}W_{1}\left(\tau \right){\left(t-\tau \right)}^{\alpha -1}d\tau ,\\ & & E_{0}+\frac{\beta }{\gamma \Gamma \left(\alpha \right)}\int_{0}^{t}\tau^{\alpha -1}W_{2}\left(\tau \right){\left(t-\tau \right)}^{\alpha -1}d\tau ,\\ & & I_{0}+\frac{\beta }{\gamma \Gamma \left(\alpha \right)}\int_{0}^{t}\tau^{\alpha -1}W_{3}\left(\tau \right){\left(t-\tau \right)}^{\alpha -1}d\tau ,\\ & & R_{0}+\sigma \frac{\beta }{\gamma \Gamma \left(\alpha \right)}\int_{0}^{t}\tau^{\alpha -1}W_{4}\left(\tau \right){\left(t-\tau \right)}^{\alpha -1}d\tau ,\\ & & V_{0}+\upsilon \frac{\beta }{\gamma \Gamma \left(\alpha \right)}\int_{0}^{t}\tau^{\alpha -1}W_{5}\left(\tau \right){\left(t-\tau \right)}^{\alpha -1}d\tau ), \end{array}$$

which has a unique fixed point when

$$\frac{℘\beta \kappa \Sigma }{\gamma \Gamma \left(\alpha \right)}\langle 1,℘\rangle 0,\alpha >0,\beta >0$$

for all contraction and continuous variables.

Next, we illustrate some examples to apply Theorems 1 and 2.

Example 5 Let ρ=0,υ=σ=1 with α=0.8,β=0.38, we have

(6) $$\begin{array}{ccc} & & {}^{ARL}D_{t}^{0.8,0.38}S\left(t\right)=-S\left(t\right)\\ & & {}^{ARL}D_{t}^{0.8,0.38}E\left(t\right)=-E\left(t\right)\\ & & {}^{ARL}D_{t}^{0.8,0.38}I\left(t\right)=\varrho E\left(t\right)-I\left(t\right)\\ & & {}^{ARL}D_{t}^{0.8,0.38}R\left(t\right)=I\left(t\right)\\ & & {}^{ARL}D_{t}^{0.8,0.38}V\left(t\right)=S\left(t\right), \end{array}$$

with

$$\frac{℘\beta \kappa \Sigma }{\Gamma \left(\alpha \right)}=\frac{1}{2}<1,$$

where

• $\Sigma =\frac{3}{2}T^{0.6};$
• κ=1;
• $℘=\frac{2}{3T^{0.6}}$.

Hence, System (6) has a unique solution for all t∈[0,T] (Theorem 1).

Example 6 Let ρ=0,υ=σ=1 with α=0.8,β=0.38,γ=2, we have

(7) $$\begin{array}{ccc} & & {}^{ARL}D_{t}^{0.8,0.38,2}S\left(t\right)=-S\left(t\right)\\ & & {}^{ARL}D_{t}^{0.8,0.38,2}E\left(t\right)=-E\left(t\right)\\ & & {}^{ARL}D_{t}^{0.8,0.38,2}I\left(t\right)=\varrho E\left(t\right)-I\left(t\right)\\ & & {}^{ARL}D_{t}^{0.8,0.38,2}R\left(t\right)=I\left(t\right)\\ & & {}^{ARL}D_{t}^{0.8,0.38,2}V\left(t\right)=S\left(t\right), \end{array}$$

with

$$\frac{℘\beta \kappa \Sigma }{\gamma \Gamma \left(\alpha \right)}=\frac{1}{4}<1,$$

where

• $\Sigma =\frac{3}{2}T^{0.6};$
• κ=1;
• $℘=\frac{2}{3T^{0.6}}$.

Thus, System (7) has only one solution for all t∈[0,T] (Theorem 2).

## Limitations and challenges

An overview of the model's strengths documents the regional and temporal fluctuations in immunization campaigns. Models the delayed effects and memory effects of vaccinations accurately. more effectively manages intricate, irregular vaccination patterns than both integer-order and traditional fractional models. Increases forecast accuracy for the persistence and eradication of outbreaks. Constraints: high processing expense for extensive simulations. Reliant on data: High-quality data is necessary for precise parameter estimation. sensitive to ambiguity in background information and immunization histories. The biological interpretation of fractional and fractal characteristics is complicated.

**Table:** 

Chalenges	Solutions
Training	Regularization, preconditioning, adaptive precision
Truncation errors	Refined time discretization, higher-order schemes, adaptive time-stepping
Computational complexity	Sparse matrix techniques, fast algorithms, parallel computing
Stability of time-stepping schemes	Implicit methods, stability analysis, fractional multi-step methods
Boundary and initial conditions	Extended initial conditions, compatibility conditions
Sensitivity to fractional order	Sensitivity analysis, robust discretization
Data-Associated Difficulties	Prioritize enhancing data gathering and incorporating data from several sources (e.g., ecological surveys, GPS tracking). Create effective mathematical techniques to lower computing expenses. Collaborate with public health specialists, ecologists, and epidemiologists to improve the clarity and utility of the methodology.

## Ethics statements

Not applicable.

## Declaration of competing interest

The authors declare that they have no known competing financial interests or personal relationships that could have appeared to influence the work reported in this paper.

## Data Availability

No data was used for the research described in the article.
